# P-1165. Development of Novel CD4+ Cell Targeting Antibody-Drug Conjugates for HIV Treatment

**DOI:** 10.1093/ofid/ofaf695.1358

**Published:** 2026-01-11

**Authors:** Chin-Ming Chang, Jason mao, Gina C L Wen

**Affiliations:** TaiMed Biologics, Taipei City, Taipei, Taiwan; TaiMed Biologics, Taipei City, Taipei, Taiwan; TaiMed Biologics, Taipei City, Taipei, Taiwan

## Abstract

**Background:**

Antibody-drug conjugates (ADCs) represent a promising therapeutic modality, combining the specificity of antibodies with the potency of small-molecule drugs. TMB-365 is a long-acting, broadly-blocking antibody improved from the commercialized ibalizumab. It is an effective post-attachment entry inhibitor that preventing HIV-1 from entering the CD4-positive cells by attaching to the CD4 receptors on their surface. This study reports the development and evaluation of a novel ADC, TMB-365-015, designed for HIV treatment.

**Figure:**
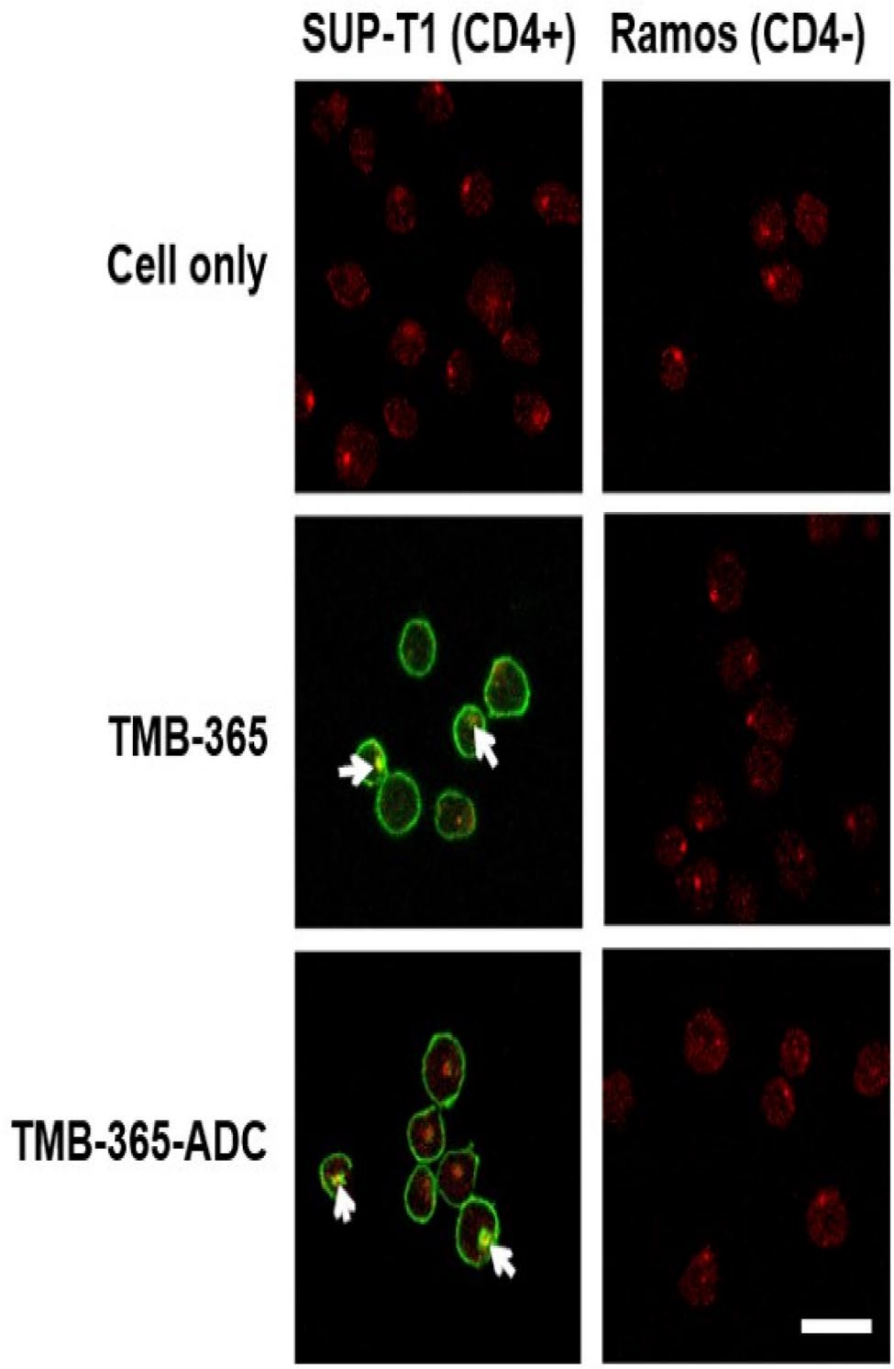
Internalization analysis of TMB-365 and ADC by confocal microscopy. Green: TMB-365 or TMB-365-ADC (anti-IgG); red: lysosome (LAMP1); white arrow: co-localization of TMB-365 or ADC with LAMP1-stained lysosomes (Yellow). Scale bar: 20 μm.

**Figure:**
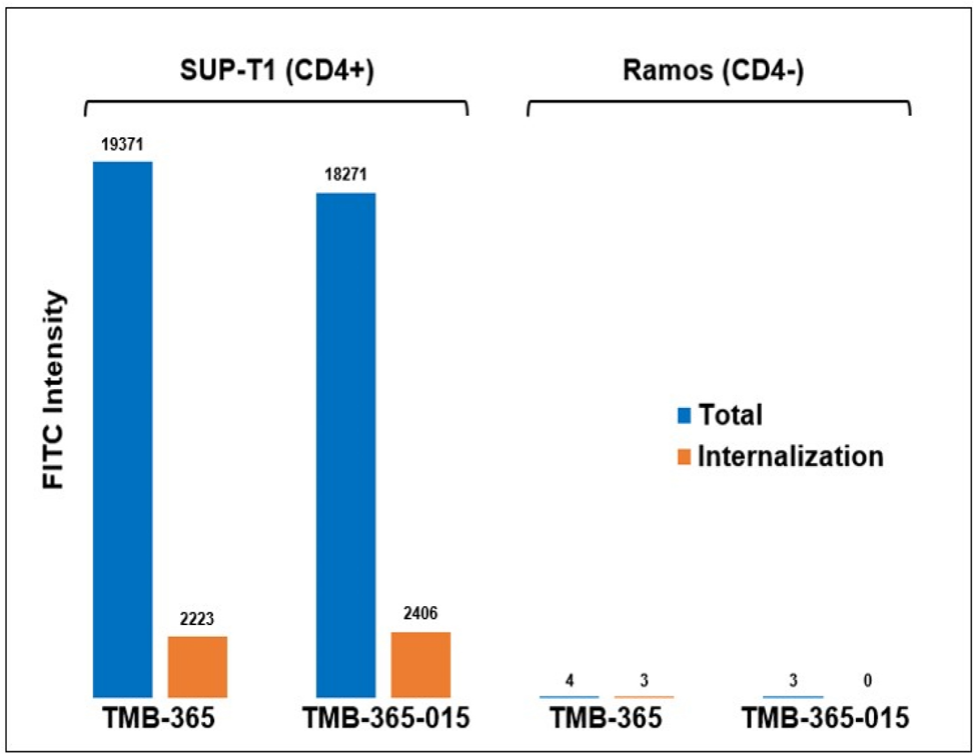
Internalization analysis of TMB-365 and TMB-365-015 in SUP-T1 and Ramos cells by flow cytometry.

**Methods:**

TMB-365 was conjugated with INI-HIV-015 (derivative of integrase inhibitor, INI) as a payload by cysteine conjugation. Conjugation parameters were optimized to achieve target quality attributes, including drug-to-antibody ratio (DAR) and low levels of high molecular weight species (HMWS). The cellular internalization of the ADC and in vitro intracellular drug release were evaluated in CD4-positive (SUP-T1) and CD4-negative (Ramos) cell lines using confocal microscopy, flow cytometry and LC-MS/MS. Additionally, the stability of the linker-payload was assessed in human plasma before conjugation.

**Figure:**
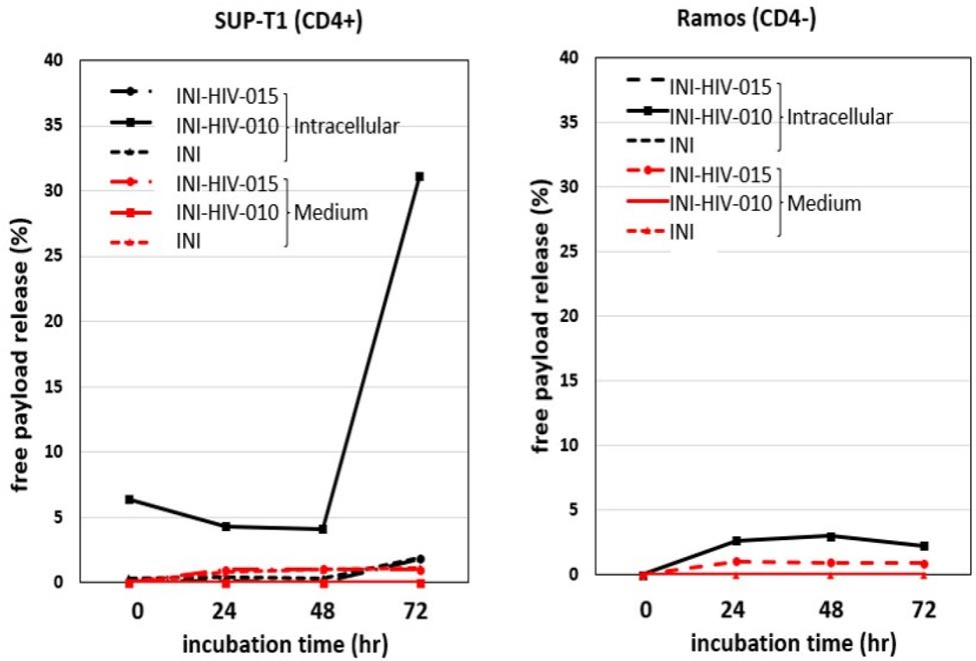
Release (%) of INI-HIV-015, INI-HIV-010, and integrase inhibitor (INI) from TMB-365-015 in SUP-T1, Ramos Cells and culture medium over 72 hours.

**Results:**

TMB-365-015 was successfully synthesized with a DAR of 4 and HMWS < 1.5%. The ADC demonstrated stability at 4°C for at least two weeks and following one freeze-thaw cycle. Stability studies of the linker-payload, INI-HIV-010, in human plasma showed > 90% integrity after 16 hours. TMB-365-015 ADC exhibited specific internalization into SUP-T1 cells (Figure 1), with minimal uptake in Ramos cells (Figure 2), indicating CD4-dependent targeting. Intracellular drug release studies showed that the release of INI and INI-HIV-010 (the metabolite of INI-HIV-015) from TMB-365-015 ADC ranged from 1.90% to 31.10% over 72 hours, with higher release observed in SUP-T1 cells (Figure 3).

**Conclusion:**

The novel ADC, TMB-365-015, was successfully produced and demonstrated favorable in vitro properties, including target cell selectivity, intracellular drug release, and stability. These results support the potential of this ADC as a novel therapeutic strategy for HIV treatment.

**Disclosures:**

Chin-Ming Chang, Ph.D., TaiMed Biologics: Board Member|TaiMed Biologics: Stocks/Bonds (Public Company) Jason mao, Ph.D., TaiMed Biologics: Stocks/Bonds (Public Company) Gina CL Wen, PhD, TaiMed Biologics: Stocks/Bonds (Public Company)

